# The Combined Value of the Prognostic Nutritional Index and Pan‐Immune‐Inflammation Value in Predicting Neoadjuvant Chemotherapy and Survival in Locally Advanced Colon Cancer: A Retrospective Study

**DOI:** 10.1002/iid3.70572

**Published:** 2026-11-05

**Authors:** Zihao Chen, Jiangyu Li, Ruoxi Gao, Lei Liang, Jun Yang, Ting Zhao

**Affiliations:** ^1^ Department of Surgical Oncology The First Affiliated Hospital of Kunming Medical University Kunming Yunnan China; ^2^ Medical College Kunming University Kunming Yunnan China; ^3^ Basic Medical College Kunming Medical University Kunming Yunnan China; ^4^ Department of Clinical Pharmacy The First Affiliated Hospital of Kunming Medical University Kunming Yunnan China

**Keywords:** locally advanced colon cancer, neoadjuvant chemotherapy, pan‐immune‐inflammation value, prognosis, prognostic nutritional index, treatment response

## Abstract

**Background:**

The pan‐immune inflammation value (PIV) and prognostic nutritional index (PNI) are established markers of systemic inflammatory/immune status and nutritional status, respectively. The potential synergistic effect of combining these indices remains unexplored. This study aimed to evaluate the utility of a novel combined PIV‐PNI score for predicting chemosensitivity and survival outcomes in patients with locally advanced colon cancer (LACC) undergoing neoadjuvant chemotherapy (NACT), within a cohort from the Yunnan Plateau region.

**Methods:**

We conducted a retrospective analysis of 241 LACC patients who received NACT followed by radical resection. Tumor response was classified according to AJCC/CAP guidelines, with patients stratified into a TRG3 (minimal or no pathological response) group (*n* = 40) and a non‐TRG3 group (*n* = 201). Pre‐treatment PIV and PNI were measured, with optimal cut‐off values identified by receiver operating characteristic (ROC) curve analysis. The PIV‐PNI score was calculated as follows: a score of 2 indicated high PIV and low PNI; a score of 1 indicated either high PIV or low PNI alone; and a score of 0 indicated neither.

**Results:**

The PIV‐PNI score was significantly higher in the TRG3 group compared to the non‐TRG3 group (*p* < 0.0001). The optimal cut‐off values for PIV and PNI via ROC analysis were ≥ 218.7 and ≤ 50.6, respectively. A higher PIV‐PNI score was associated with inferior survival outcomes. Multivariate analysis identified the PIV‐PNI score as an independent prognostic factor for both overall survival and disease‐free survival.

**Conclusions:**

The PIV‐PNI score, a low‐cost and readily available composite index, demonstrates significant clinical utility as a predictive marker for tumor response to NACT and prognosis in LACC patients.

**Novelty and Impact of the Work:**

PIV–PNI score, which integrates systemic inflammation and immunonutritional status, demonstrates strong predictive value for chemosensitivity and long‐term survival, offering a clinically accessible tool to guide personalized treatment strategies in the neoadjuvant setting.

**Abbreviations**
AUC: area under the curve
DFS: disease‐free survival
LACC: locally advanced colon cancer
NLR: neutrophil‐to‐lymphocyte ratio
OS: overall survival
PIV: pan‐immune inflammation value
PLR: platelet‐to‐lymphocyte ratio
PNI: prognostic nutritional index
ROC: receiver operating characteristic
SII: systemic immune‐inflammation index

## 1 Introduction

Colorectal cancer (CRC) represents a major global health challenge, with its incidence and mortality rates continuing to rise [1]. In China, this disease accounts for a substantial proportion of cancer‐related deaths, and a majority of new cases are diagnosed at a locally advanced stage. For patients with locally advanced colon cancer (LACC), the current standard of care typically involves surgical resection, which offers the best potential for cure [2]. The role of neoadjuvant chemotherapy (NACT) has gained increasing recognition in this setting, as evidenced by clinical trials demonstrating its ability to reduce tumor burden, facilitate complete surgical resection, and ultimately improve survival outcomes [3]. Regimens such as CAPEOX (capecitabine and oxaliplatin) and FOLFOX (fluorouracil, leucovorin, and oxaliplatin) have become established as effective and safe NACT options for LACC [4, 5].

However, a significant clinical challenge remains, as approximately 40% of LACC patients derive little or no benefit from NACT. For these patients, exposure to chemotherapy can lead to unnecessary treatment toxicity, delayed definitive surgery, and increased economic burden, without any corresponding therapeutic gain [6]. The current ability to predict which patients will respond favorably to NACT is limited [7]. Although clinical parameters such as age, TNM stage, Eastern Cooperative Oncology Group (ECOG) performance status, and body mass index (BMI) are sometimes used for pretreatment assessment, their predictive power is suboptimal [8]. The histopathological tumor regression grade (TRG), while considered the reference standard for evaluating chemosensitivity, can only be determined postoperatively, limiting its utility for pretreatment decision‐making [9]. Therefore, identifying readily available and reliable biomarkers that can preoperatively stratify patients according to their likelihood of responding to NACT is a crucial unmet need in clinical management [10, 11].

The relationship between systemic inflammation, immune status, and cancer progression is well documented [12, 13]. This has led to interest in hematologic indices as potential biomarkers. Established parameters such as the systemic immune‐inflammation index (SII), neutrophil‐to‐lymphocyte ratio (NLR), and platelet‐to‐lymphocyte ratio (PLR) have shown prognostic value in various malignancies. More recently, pan‐immune inflammation value (PIV), which integrates counts of neutrophils, platelets, monocytes, and lymphocytes, has emerged as a more comprehensive reflector of the host immune and inflammatory state, demonstrating prognostic utility in several advanced cancers [14]. Similarly, prognostic nutritional index (PNI), derived from serum albumin and lymphocyte count, serves as an indicator of both immunocompetence and nutritional status. A low PNI has been consistently associated with poorer outcomes and reduced chemosensitivity in cancers, including gastric cancer and non‐small cell lung cancer [10].

Given that PIV and PNI reflect distinct but interrelated aspects of the host‐tumor interaction—systemic inflammation and immunonutritional status, respectively—their combination may yield a more robust predictive tool. However, the potential of a composite PIV‐PNI score to predict outcomes specifically in LACC patients receiving NACT has not been explored. This study, therefore, aims to investigate whether the pretreatment PIV‐PNI score can serve as a practical biomarker for predicting pathological response to NACT and long‐term survival in a cohort of LACC patients from the Yunnan Plateau region. We also sought to determine the optimal cutoff values for this novel index and assess its correlation with clinicopathological characteristics.

## 2 Materials and Methods

### 2.1 Participants

This retrospective study enrolled 241 patients with LACC who underwent NACT at the First Affiliated Hospital of Kunming Medical University between January 2014 and May 2019 [15]. The inclusion criteria were: (1) pathologically confirmed colon adenocarcinoma; (2) preoperative abdominal computed tomography (CT) staging of T2‐T4N + M0; (3) age 18 years or older; (4) no prior antitumor therapy; (5) ECOG performance status ≤ 2 and adequate cardiac, pulmonary, and renal function to tolerate chemotherapy; and (6) absence of active infection or acute/chronic inflammatory conditions prior to surgery. Exclusion criteria comprised: (1) R1 or R2 resection; (2) history of other malignancies; (3) concurrent acute infection, autoimmune disorder, or hematologic disease; (4) incomplete clinical data (cases with missing baseline laboratory values were excluded from the analysis); and (5) deviation from the prescribed NACT regimen. Standard NACT consisted of two cycles of CAPEOX or FOLFOX, a duration selected to balance early response evaluation with surgical timing in accordance with NCCN guidelines. Peripheral blood samples were obtained from all patients within 1 week prior to the initiation of NACT to establish baseline values. The study was conducted in accordance with the Declaration of Helsinki and approved by the Institutional Ethics Committee (Approval No.: Law Review No. 124). Written informed consent was obtained from all participants prior to treatment, and patient data were handled confidentially without any intervention in clinical management.

### 2.2 Calculation of Inflammatory and Nutritional Indices

The PIV was calculated using the formula: PIV = [neutrophil count (×10^9^/L) × platelet count (×10^9^/L) × monocyte count (×10^9^/L)]/lymphocyte count (×10^9^/L). The PNI was computed as: PNI = albumin (g/L) + 5 × lymphocyte count (×10^9^/L). Optimal cut‐off values for PIV and PNI were determined using receiver operating characteristic (ROC) curve analysis to discriminate between TRG3 and non‐TRG3 responders. A composite PIV‐PNI score was then constructed as follows: Score 2: high PIV (≥ cut‐off) and low PNI (≤ cut‐off); Score 1: either high PIV or low PNI; Score 0: low PIV and high PNI.

### 2.3 NACT Regimens

Among the initially screened 526 colon adenocarcinoma patients, 269 (51.2%) received the CAPEOX regimen, and 257 (48.8%) received FOLFOX. The CAPEOX regimen consisted of intravenous oxaliplatin (130 mg/m^2^) on Day 1, plus oral capecitabine (1000 mg/m^2^ twice daily) for 14 days. The FOLFOX regimen comprised oxaliplatin (85 mg/m^2^) and calcium folinate (400 mg/m^2^) on Day 1, followed by bolus fluorouracil (400 mg/m^2^) on Day 1 and continuous infusion (1200 mg/m^2^/day) on Days 2–3. Each patient completed two cycles of NACT. Treatment‐related toxicities were graded according to the Common Terminology Criteria for Adverse Events (CTCAE) version 5.0.

### 2.4 Assessment of Treatment Response

Tumor response was evaluated using contrast‐enhanced abdominal CT performed before and 3 weeks after completion of NACT. Patients ingested 800–1000 mL of water 30 min prior to scanning (unless contraindicated), and intramuscular scopolamine butylbromide was administered to reduce gastrointestinal motility and improve gastric distention. Target lesions were identified and measured by a senior radiologist; the longest diameter was recorded for non‐nodal lesions, and the short‐axis diameter was recorded for lymph nodes (> 1.5 cm). The sum of diameters from all target lesions served as the baseline for subsequent comparisons. Treatment response was classified according to Response Evaluation Criteria in Solid Tumors (RECIST) v1.1 as complete response (CR), partial response (PR), stable disease (SD), or progressive disease. Objective response rate (ORR) included CR + PR, and disease control rate included CR + PR + SD. Pathological response was evaluated by two independent pathologists using the American Joint Committee on Cancer/College of American Pathologists (AJCC/CAP) TRG system: TRG0 (no residual tumor), TRG1 (minimal residual tumor), TRG2 (moderate response), and TRG3 (minimal or no response). For analysis, patients were dichotomized into TRG3 (poor response) and non‐TRG3 (TRG0‐2) groups.

### 2.5 Follow‐Up

Overall survival (OS) was defined as the time from treatment initiation to cancer‐related death or last follow‐up [16]. Disease‐free survival (DFS) referred to the time from randomization to disease recurrence, progression, or death. Postoperative surveillance included abdominal contrast‐enhanced CT every 3 months for the first 3 years and every 6 months thereafter. Follow‐up data were collected via telephone, outpatient visits, and hospital records.

### 2.6 Statistical Analysis

Statistical analyses were performed using GraphPad Prism version 10.1.1 (GraphPad Software, San Diego, CA). Normality of continuous variables was assessed using the Shapiro–Wilk test; normally distributed data were expressed as mean ± standard deviation, and non‐normal data as median (interquartile range). Group comparisons were made using the independent *t*‐test or Mann‐Whitney *U*‐test, as appropriate. Optimal cut‐off values for PIV and PNI were determined via ROC analysis based on differentiation of TRG3 status, specifically identified using the maximum Youden index (sensitivity + specificity − 1). The area under the curve (AUC) was used to compare predictive performance. Survival curves were generated using the Kaplan–Meier method and compared with the log‐rank test. Univariate and multivariate Cox proportional hazards models were employed to identify independent prognostic factors, with results reported as hazard ratios (HR) and 95% confidence intervals (CIs). Correlation between indices was assessed using Spearman's rank correlation. A two‐sided *p* value < 0.05 was considered statistically significant.

## 3 Results

### 3.1 Patient Demographic and Tumor Characteristics

This study enrolled 241 patients with LACC. The cohort comprised 163 males (67.6%) and 78 females (32.4%), with a minority ethnic representation of 8.7% (21 patients) and a Han Chinese majority of 91.3% (220 patients). Clinical characteristics and laboratory parameters are summarized in Table S1. The age at diagnosis ranged from 24 to 83 years, with a median of 59 years. The mean BMI for the entire cohort was 22.5 ± 3.1 kg/m^2^. According to the American Society of Anesthesiologists Physical Status classification, 195 patients were Grade I, and 46 were Grade II, with no patients classified as Grade III or higher. All patients received two cycles of NACT, with 122 patients (50.6%) receiving the CAPEOX regimen and 119 patients (49.4%) receiving the FOLFOX regimen (Figure 1). Subsequent radical D2 surgical resection was performed on all patients, consisting of 4 left hemicolectomies (1.7%), 1 transverse colectomy (0.4%), 17 right hemicolectomies (7.0%), and 219 sigmoid colectomies (90.9%). Laparoscopic resection was the predominant surgical approach, performed in 226 patients (93.8%), while the remaining 15 patients (6.2%) underwent open surgery. The mean PIV and PNI prior to NACT were 380.7 ± 645.6 (range: 27.8–2636.7) and 51.4 ± 8.8 (range: 31.7–81.1), respectively. A significant inverse correlation was observed between these two indices (*r* = −0.2468, *p* < 0.0001; Figure 2A).

**Figure 1:**
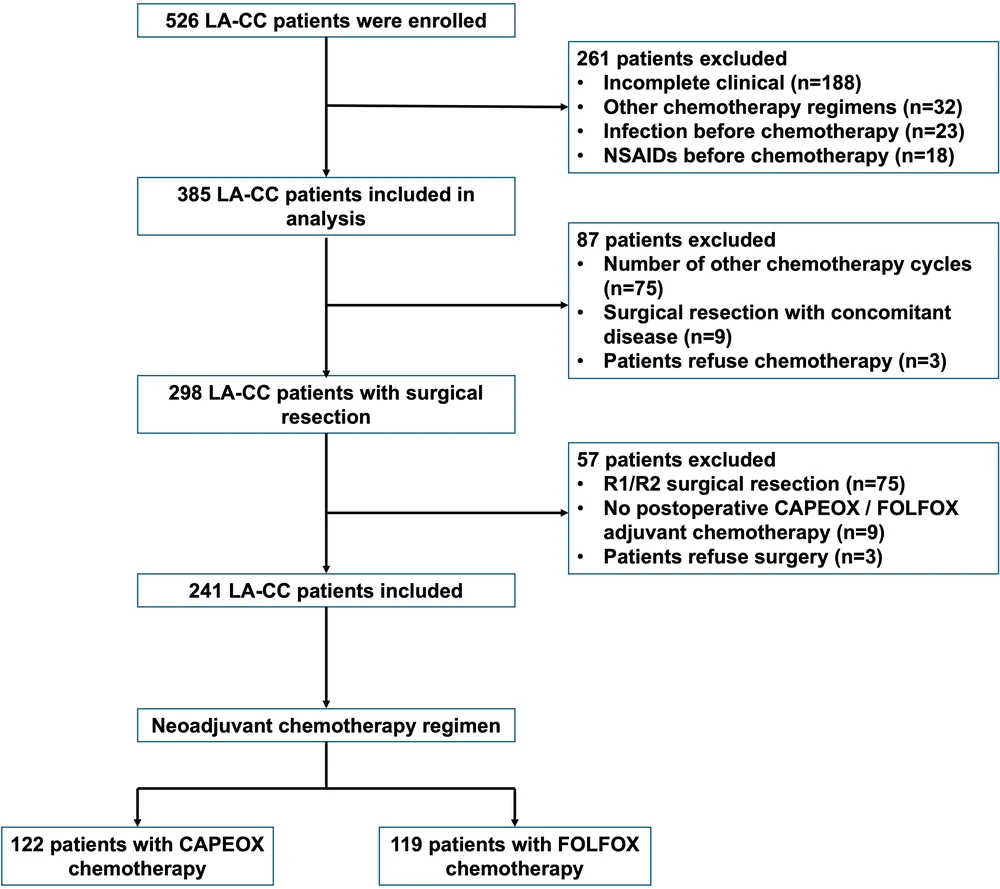
Flow chart of patient enrollment and exclusion.

**Figure 2:**
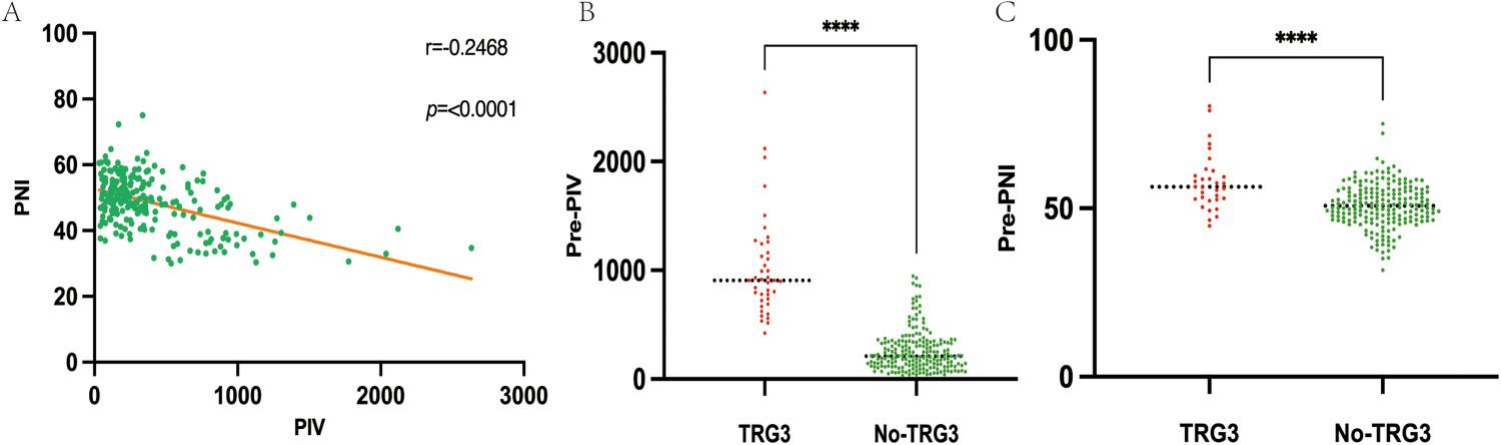
Relationship between TRG3/non‐TRG3 and PIV/PNI. (A) Correlation analysis between PIV and PNI; (B) PIV values of TRG3/non‐TRG3 patients; (C) PNI values of TRG3/non‐TRG3 patients.

### 3.2 Pathological Response and Association With PIV/PNI

Following NACT completion, all patients underwent radical surgery. Pathological assessment using the TRG system revealed: TRG 0 in 38 patients (15.8%), TRG 1 in 74 patients (30.7%), TRG 2 in 89 patients (36.9%), and TRG 3 in 40 patients (16.6%). Comparative analysis demonstrated that patients with TRG 3 had significantly higher pre‐NACT PIV (626.4 ± 1342.52 vs. 332.5 ± 336.4, *p* < 0.0001) and significantly lower pre‐NACT PNI (50.3 ± 7.0 vs. 51.6 ± 13.0, *p* < 0.0001) compared to non‐TRG 3 patients (Figure 2B,C). ROC curve analysis was employed to determine the optimal cutoff values for PIV and PNI for discriminating TRG 3 from non‐TRG 3 patients. Pre‐NACT PIV demonstrated a sensitivity of 85.1% and specificity of 75.0% (AUC = 0.8873, 95% CI: 0.8308–0.9437, *p* < 0.0001). Pre‐NACT PNI showed a sensitivity of 93.5% and specificity of 62.5% (AUC = 0.8434, 95% CI: 0.7698–0.9170, *p* < 0.0001) (Figure 3A,B).

**Figure 3:**
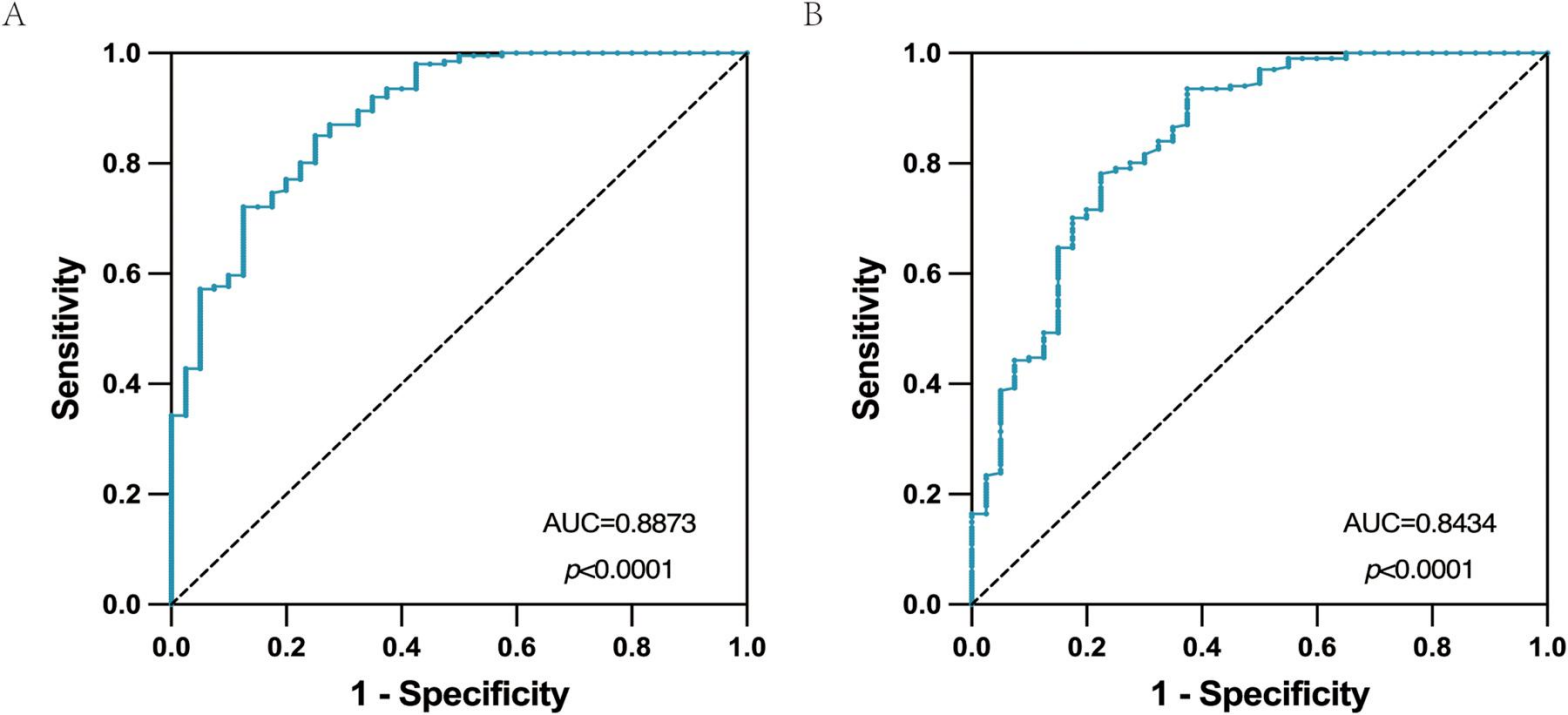
ROC curves for discriminating patients with TRG 3 and those with non‐TRG 3 according to values of the PIV (A) and PNI (B).

### 3.3 The PIV‐PNI Scoring System and Its Clinicopathological Correlations

A composite PIV‐PNI score was defined as follows: a score of 2 indicated a PIV above the cutoff (≥ 218.7) and a PNI below the cutoff (≤ 50.6); a score of 1 indicated meeting one of these criteria; a score of 0 indicated neither. Distribution among patients was: 72 (29.9%) with score 0, 96 (39.8%) with score 1, and 73 (30.3%) with score 2. Clinicopathological analysis revealed that a PIV‐PNI score of 0 was associated with a higher proportion of well/moderately differentiated tumors, whereas a score of 2 correlated with a predominance of poorly differentiated histology (*p* = 0.0117). A significant association was also observed with the surgical approach; all patients with a score of 0 underwent laparoscopic surgery, and the likelihood of open surgery increased progressively with higher scores (*p* = 0.0031). No other significant correlations with clinicopathological features were identified (*p* > 0.05) (Table S2).

### 3.4 Treatment Response and Toxicity

All patients received standard‐dose NACT. Treatment intensity was reduced in only 3 patients (1.2%) due to Grade 3 adverse events (AEs, CTCAE v5.0), primarily nausea and vomiting, which resolved with supportive care. An additional 36 patients (14.9%) experienced Grades 1–2 AEs, mostly gastrointestinal, managed symptomatically without dose modification. Tumor response was evaluated per RECIST 1.1 after two NACT cycles. In the CAPEOX group (*n* = 122), responses were: 14 CR, 70 PR, and 38 SD (Figure 4A). In the FOLFOX group (*n* = 119), responses were: 11 CR, 70 PR, and 38 SD (Figure 4B). No disease progression was recorded in either group. A clear inverse relationship was observed between the PIV‐PNI score and the ORR, with the highest ORR in the score 0 group and the lowest in the score 2 group (Table S3).

**Figure 4:**
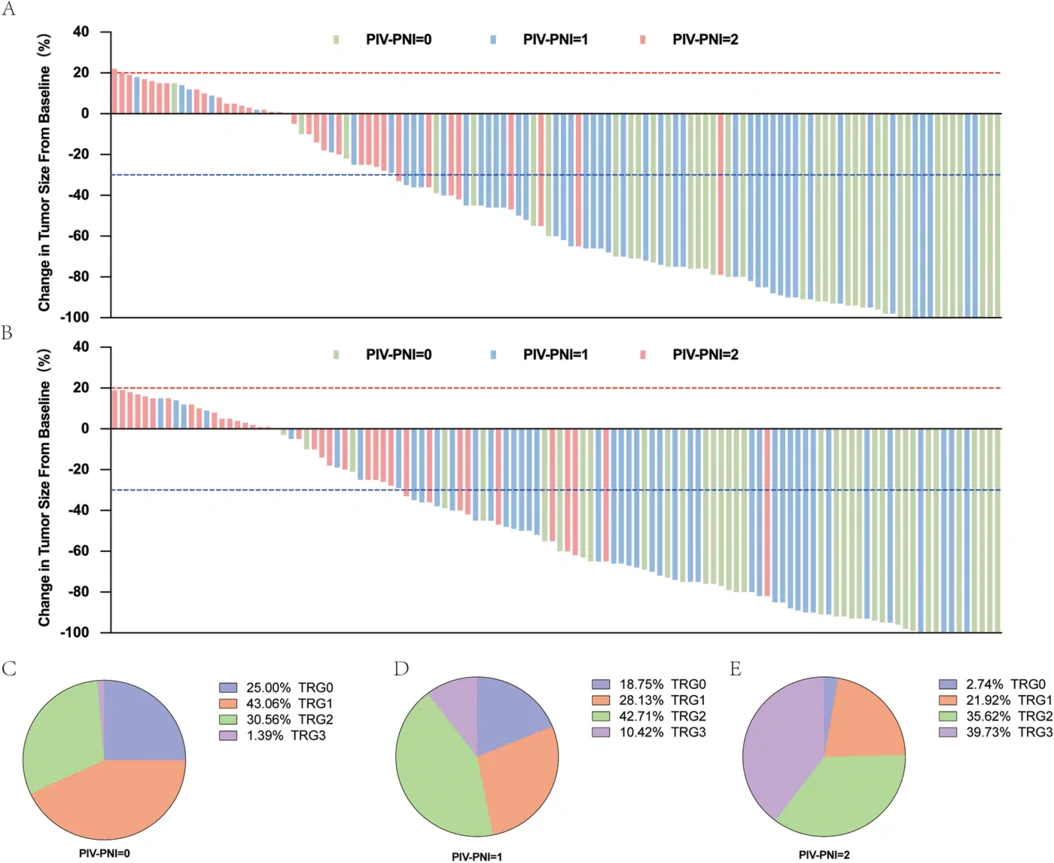
The ratios of imaging assessment changes to postoperative pathological TRG responses among different PIV‐PNI score groups. (A) CT imaging changes after 2 chemotherapy cycles based on the CAPEOX preoperative neoadjuvant chemotherapy regimen, evaluated using the RECIST 1.1 standard; (B) CT imaging changes after 2 chemotherapy cycles based on the FOLFOX preoperative neoadjuvant chemotherapy regimen, also evaluated using the RECIST 1.1 standard; (C) Distribution of postoperative pathological TRG grades in patients with PIV‐PNI score of 0; (D) Distribution of postoperative pathological TRG grades in patients with PIV‐PNI score of 1; (E) Distribution of postoperative pathological TRG grades in patients with PIV‐PNI score of 2.

### 3.5 Surgical Outcomes and Pathological Regression by PIV‐PNI Score

The overall incidence of post‐operative complications was 12.03% (29/241), including: pneumonia (16 cases, 6.64%), anastomotic leakage (4 cases, 1.66%), intestinal obstruction (4 cases, 1.66%), lower limb deep vein thrombosis (3 cases, 1.24%), and intra‐abdominal hemorrhage (2 cases, 0.83%). According to the Clavien‐Dindo classification, complications were Grade I in 20 patients, Grade II in 7, and Grade III in 2. Pathological response was strongly associated with the PIV‐PNI score (*p* < 0.0001). The proportion of patients achieving TRG 0–2 (non‐TRG 3) increased as the PIV‐PNI score decreased (Table S3). Specifically, for score 0: TRG 0: 25.00% (18), TRG 1: 43.06% (31), TRG 2: 30.56% (22), TRG 3: 1.39% (1) (Figure 4C). For score 1: TRG 0: 18.75% (18), TRG 1: 28.13% (27), TRG 2: 42.71% (41), TRG 3: 10.42% (10) (Figure 4D). For score 2: TRG 0: 2.74% (2), TRG 1: 21.92% (16), TRG 2: 35.62% (26), TRG 3: 39.73% (29) (Figure 4E).

### 3.6 Prognostic Impact of the PIV‐PNI Score

Kaplan–Meier analysis revealed significantly different 5‐year OS across PIV‐PNI score groups (all *p* < 0.05, Figure 5A). The 5‐year DFS rates were 53.27%, 34.15%, and 15.15% for scores of 0, 1, and 2, respectively, with statistically significant differences between groups (all *p* < 0.05, Figure 5B). Cox multivariate analysis identified the following as independent risk factors for 5‐year OS: BMI (HR = 0.8890, 95% CI: 0.7949–0.9936, *p* = 0.0385), Tumor Invasion Depth (HR = 0.5310, 95% CI: 0.3146–0.8923, *p* = 0.0167), Lymph Node Metastasis (HR = 0.4369, 95% CI: 0.2076–0.8328, *p* = 0.0183), Clinical Stage (HR = 8.683, 95% CI: 3.506–23.000, *p* < 0.0001), PIV‐PNI Score (HR = 2.287, 95% CI: 1.433–3.792, *p* = 0.0008), and Tumor Response (HR = 1.765, 95% CI: 1.068–2.921, *p* = 0.0258). Independent risk factors for 5‐year DFS were: Minority Ethnicity (HR = 3.050, 95% CI: 1.057–8.799, *p* = 0.0392), Clinical Stage (HR = 5.495, 95% CI: 2.475–12.650, *p* < 0.0001), Surgical Procedure (HR = 0.2333, 95% CI: 0.0995–0.6021, *p* = 0.0014), and PIV‐PNI Score (HR = 2.217, 95% CI: 1.475–3.448, *p* = 0.0002). The NACT regimen was not a significant prognostic factor for either OS or DFS (*p* = 0.8723 and *p* = 0.8934, respectively) (Table 1).

**Figure 5:**
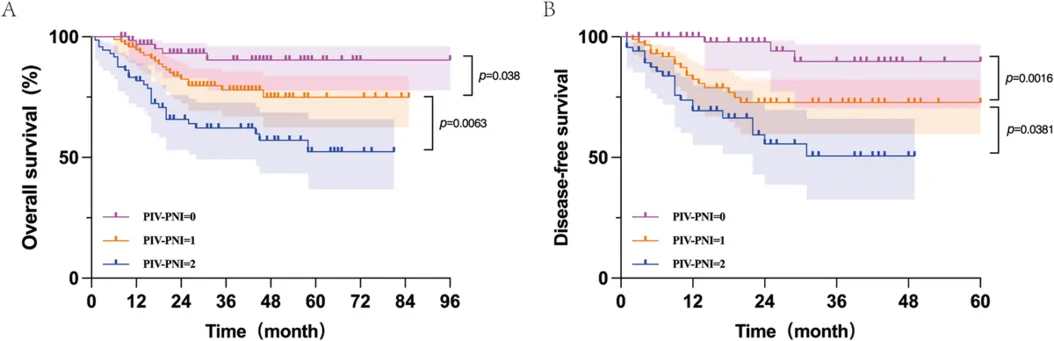
Kaplan–Meier survival curves in patients with LACC. (A) 5‐year overall survival based on PIV‐PNI score; (B) 5‐year disease‐free survival based on PIV‐PNI score.

**Table 1:** Multivariate analysis of the clinicopathological characteristics for the prognosis of LACC patients.

Variables	5‐year OS multivariate analysis			5‐year DFS multivariate analysis			
	Hazard ratio	95% CI	*p* value	Hazard ratio		95% CI	*p* value
Gender	0.9505	0.4205–2.032	0.8986	1.040		0.5227–2.004	0.9088
Age	1.005	0.9759–1.036	0.7391	1.002		0.9764–1.028	0.8982
Minority ethnic group	0.9140	0.04911–4.909	0.9326	3.050		1.057–8.799	0.0392
BMI	0.8890	0.7949–0.9936	0.0385	0.9376		0.8519–1.032	0.1872
MMR status	1.861	0.5159–5.320	0.2845	0.7349		0.1732–2.118	0.6178
Tumor size (cm)	1.085	0.9124–1.268	0.3241	0.9915		0.8348–1.155	0.9171
Infiltrating depth	0.5310	0.3146–0.8923	0.0167	0.7026		0.4336–1.125	0.1450
Lymph node metastasis	0.4369	0.2076–0.8328	0.0183	0.7240		0.3938–1.276	0.2793
Clinical stage	8.683	3.506–23.00	< 0.0001	5.495		2.475–12.65	< 0.0001
Differentiation degree	0.4719	0.1908–1.284	0.1192	1.559		0.6124–4.586	0.3814
Surgical procedures	0.6313	0.2363–1.927	0.3844	0.2333		0.0995–0.6021	0.0014
Chemotherapy	1.013	0.883–1.991	0.8723	1.021		0.821–1.996	0.8934
PIV‐PNI score	2.287	1.433–3.792	0.0008	2.217		1.475–3.448	0.0002
Tumor response	1.765	1.068–2.921	0.0258	1.330		0.7925–2.251	0.2825

Abbreviations: BMI, body mass index; DFS, disease‐free survival; LACC, locally advanced colon cancer; MMR, mismatch repair‌; OS, overall survival; PIV, pan‐immune‐inflammation value; PNI, Prognostic nutritional index.

## 4 Discussion

In recent years, systemic inflammatory responses and host nutritional status have been increasingly recognized as critical determinants influencing tumor progression, therapeutic response, and long‐term outcome. Inflammatory mediators can remodel the tumor microenvironment and modulate immune function, thereby promoting tumor invasion, angiogenesis, and metastasis, whereas nutritional parameters serve as indirect reflections of the host's immune competence and metabolic reserve [17, 18]. Integrating these two dimensions provides a more comprehensive understanding of host‐tumor interactions [19]. In our previous studies, we found that combined PIV and PNI could serve as a prognostic biomarker for CRC undergoing enterectomy, mainly for patients with colon cancer who did not receive chemotherapy before the surgery [20]. In the present study, we focused on patients with LACC. The combined use of PIV and PNI was explored to evaluate the effect of preoperative neoadjuvant therapy and to guide the clinical assessment of chemotherapy sensitivity for specific patients. These two studies constitute complementary parts of our research series. This study is the first to integrate the PIV and PNI in a cohort from the Yunnan Plateau region to develop a novel composite scoring system—the PIV‐PNI score. This score not only predicts pathological response to NACT in patients with LACC but also serves as an independent prognostic indicator of long‐term survival following curative resection.

Inflammation is widely regarded as the “seventh hallmark of cancer” and is intricately linked to the immune landscape of the tumor microenvironment [21, 22]. Neutrophils, platelets, and monocytes can secrete pro‐tumorigenic cytokines, reactive oxygen/nitrogen species, and matrix‐degrading enzymes, all of which facilitate tumor proliferation, epithelial‐mesenchymal transition, and metastasis [23, 24]. Conversely, lymphopenia reflects impaired antitumor immunity, weakening host immune surveillance and cytotoxic responses [25]. The PIV incorporates neutrophil, platelet, monocyte, and lymphocyte counts, providing a comprehensive representation of systemic inflammation and immune dysregulation that surpasses traditional indices such as the NLR and PLR. The PNI, calculated from serum albumin and lymphocyte count, is a classic immunonutritional biomarker reflecting both nutritional reserve and immune status. Hypoalbuminemia not only indicates poor nutrition but also persistent systemic inflammation and hepatic synthetic dysfunction, while lymphocytopenia implies immune suppression [26, 27]. Consequently, combining PIV and PNI captures both inflammatory burden and nutritional‐immune reserve, allowing a multidimensional assessment of host‐tumor dynamics. By stratifying patients according to PIV‐PNI scores, this study achieved a refined physiological classification integrating inflammation and nutrition.

A pivotal finding of this study is the strong association between the PIV‐PNI score and pathological response to NACT. Patients with poor pathological regression (TRG 3) exhibited significantly higher pre‐treatment PIV‐PNI scores than those achieving TRG 0–2. Moreover, a clear gradient trend was observed: patients with a score of 0 rarely demonstrated TRG 3, whereas those with a score of 2 had a notably higher proportion of poor responders. This suggests that patients in a “hyperinflammatory‐hypoproteinemic” state before therapy exhibit decreased chemosensitivity. Concurrently, a low PNI indicates malnutrition and hypoalbuminemia, conditions that compromise tissue repair, drug metabolism, and immune effector function. The resulting lymphocyte depletion and dysfunction diminish cytotoxic capacity, impairing antitumor immunity. Collectively, these mechanisms create a proinflammatory and immunosuppressive microenvironment that favors chemoresistance and tumor progression.

NACT has become an established component of standard care for LACC, aiming to downstage tumors, increase resectability, and improve long‐term survival [28]. Nevertheless, inter‐patient variations in response remain substantial, with approximately 40% of cases showing limited benefit from conventional CAPEOX or FOLFOX regimens. Identification of predictive biomarkers for therapeutic response is therefore of paramount importance. In this cohort, the PIV‐PNI score was strongly correlated with treatment efficacy. Patients with low scores (especially 0) achieved higher objective response and pathological CR rates, whereas those with high scores (2) showed significantly lower sensitivity. Nutritional deficits reduce hepatic drug biotransformation and immune competence, further compounding therapeutic resistance [22]. Thus, a high PIV‐PNI score identifies individuals in whom both systemic inflammation and malnutrition contribute to diminished chemotherapy sensitivity.

Regarding prognosis, Kaplan–Meier analyses and multivariate Cox regression confirmed the PIV‐PNI score as an independent predictor of both OS and DFS. Patients with low PIV‐PNI scores exhibited significantly prolonged 5‑year OS and DFS compared with those scoring high, underscoring its robust prognostic value. Similar findings have been reported in gastric malignancies using the PIV‐PNI model. Chronic inflammation induces lymphocyte depletion, compromising immune‐mediated antitumor surveillance, and reduced albumin levels reflect persistent inflammation and impaired antioxidant buffering, increasing systemic vulnerability [14, 29]. Together, these processes foster aggressive tumor biology and disease recurrence.

The present findings resonate strongly with the contemporary oncologic paradigm advocating incorporation of host systemic status into prognostic models [30]. As a low‐cost, non‐invasive, and readily available composite biomarker derived from routine preoperative blood tests, the PIV‐PNI score exhibits substantial clinical translational value and actionable utility in the individualized treatment decision‐making of LACC patients receiving NACT. Pre‐therapeutically, the score can accurately stratify patients into high and low chemosensitivity subgroups: for patients with a PIV‐PNI score of 2, clinicians might hypothetically consider adjusting conventional single NACT and adopt intensified regimens or neoadjuvant chemoradiotherapy to improve tumor response rates; for score 0 patients, standard NACT may potentially be sufficient to achieve optimal downstaging, avoiding unnecessary treatment toxicity and medical burden. Perioperatively, the score guides surgical decision optimization: score 0 patients with high chemosensitivity are ideal candidates for laparoscopic minimally invasive resection, which shortens postoperative recovery and reduces complication risks; score 2 patients can receive preoperative nutritional support and low‐dose anti‐inflammatory interventions to improve treatment tolerance and surgical outcomes. Post‐operatively, the PIV‐PNI score complements the traditional TNM staging system for recurrence risk stratification: score 2 patients with high prognostic risk benefit from prolonged adjuvant chemotherapy and intensive surveillance, while score 0 patients with low risk can receive simplified surveillance and adjuvant therapy to improve quality of life. Despite these encouraging results, the molecular mechanisms underpinning the prognostic and predictive effects of the PIV‐PNI score warrant further elucidation. Proposed biological explanations include the activation of the PI3K‐AKT pathway by inflammatory cells, amplification of pro‑survival and pro‑angiogenic signals, accumulation of immunosuppressive regulatory T cells and myeloid‐derived suppressor cells, and impaired antigen presentation secondary to lymphopenia. Moreover, malnutrition‐related mitochondrial dysfunction and DNA repair deficiency increase cellular susceptibility to damage, while compromised intestinal barrier function promotes bacterial translocation and chronic inflammation, perpetuating a vicious cycle [8, 31, 32]. Altogether, the PIV‐PNI score represents an integrated reflection of inflammation, immunity, and nutrition, encapsulating the complexity of tumor‐host interactions. Despite these insights, a notable asymmetry in anatomical tumor location must be acknowledged, as right‐sided colon cancers were substantially underrepresented in our cohort (17 vs. 224). This clinical imbalance is multifactorial and reflects deep‐seated epidemiological and therapeutic realities. Epidemiologically, rectal and distal (left‐sided) colon cancers remain historically and currently predominant in the Chinese CRC population, unlike Western cohorts, where proximal tumors are more prevalent. Furthermore, NACT is routinely indicated for locally advanced rectal cancers to achieve local downstaging and facilitate sphincter preservation, whereas upfront radical surgery remains the established gold standard for most colon cancers, with NACT reserved strictly for locally unresectable or T4b disease. From a molecular oncology perspective, right‐sided tumors exhibit a significantly higher prevalence of microsatellite instability‐high or deficient mismatch repair (dMMR) status. Since dMMR tumors are notoriously resistant to conventional fluoropyrimidine‐based chemotherapy, these patients in our center are increasingly guided toward neoadjuvant immunotherapy or upfront surgery rather than standard NACT. Nevertheless, because right‐sided tumors are characterized by worse clinical outcomes, distinct systemic inflammatory responses, and unique microenvironments, the underrepresentation of this subgroup limits the generalizability of our PIV‐PNI model to proximal colon cancers. This single‐center selection bias must be noted, and future multi‐center studies with larger, balanced cohorts of right‐sided colon cancers are warranted to validate our predictive model across all anatomical subsegments.

Compared with prior studies employing the SII‐PNI scoring system in gastric cancer, this investigation is the first to propose and validate the PIV‐PNI composite model in colon cancer. The inclusion of monocyte counts in PIV enhances its capacity to characterize the immune microenvironment more comprehensively than inflammation‐only indices [33]. The PIV‐PNI score outperformed single parameters in differentiating patient risk strata and yielded greater discrimination for postoperative survival prediction, indicating superior clinical robustness and practical value. Moreover, its potential extends beyond predicting pathological response—it may serve as a useful tool in postoperative surveillance and guiding long‐term personalized management. Future research integrating the PIV‐PNI score with radiomics, molecular genetics, and transcriptomic signatures could further refine stratification and optimize individualized treatment selection. Nonetheless, these potential clinical applications are speculative and based entirely on retrospective evidence. Consequently, these findings should be interpreted as generating clinical hypotheses rather than establishing standard protocols. Future prospective randomized controlled trials are indispensable to rigorously validate the clinical utility and safety of PIV‐PNI‐guided strategies before they can be implemented into routine clinical practice.

In addition to the need for prospective validation, several other limitations of this study should be acknowledged. First, the retrospective, single‐center design is inherently prone to selection bias. Second, although the plateau population of Yunnan offers a unique cohort, it may limit the generalizability of our findings to other geographic regions or populations with different ethnic distributions. Third, several unmeasured confounders, such as baseline sub‐clinical infections, chronic comorbidities, and specific nutritional interventions during treatment, may have influenced the PIV and PNI values. Fourth, the relatively small sample size, particularly within specific ethnic subgroups, restricts the statistical power to conduct granular stratified analyses. Fifth, this study assessed inflammatory and nutritional indices at a single time point. Future prospective studies should incorporate longitudinal measurements to determine whether dynamic changes in the PIV‐PNI score are more strongly associated with treatment response and survival outcomes, as suggested by emerging research in CRC [34]. Finally, while we postulate that pathways such as PI3K‐AKT are involved, this study did not provide direct molecular evidence. Future research should prioritize prospective validation and integrate the PIV‐PNI score with molecular signatures to refine its clinical utility in the era of precision oncology and immunotherapy. To address these limitations and translate the PIV‐PNI score into clinical practice, several future research avenues are warranted. First, we plan to initiate large‐scale, multi‐center prospective clinical trials to rigorously validate the clinical utility and generalizability of the PIV‐PNI score across more diverse geographic populations and varied ethnic cohorts. Second, rather than relying solely on static baseline measurements, future studies should focus on the prospective longitudinal monitoring of PIV‐PNI alterations throughout the course of neoadjuvant therapy. Evaluating these dynamic trajectories will help clarify their real‐time predictive value, potentially allowing for adaptive treatment modifications based on a patient's evolving physiological state. Lastly, we aim to integrate the PIV‐PNI score with multi‐dimensional platforms, combining this systemic host biomarker with radiomics features, tumor genomic profiling, and immunotherapy responses. Such multi‐omics integration will facilitate the development of highly precise, comprehensive decision‐making models to optimize personalized patient stratification and therapeutic selection in the era of precision oncology.

## 5 Conclusions

In conclusion, the PIV‐PNI score emerges as a noninvasive, integrative biomarker that bridges systemic inflammation and nutritional status to inform both therapeutic responsiveness and survival outcomes in patients with LACC. Its clinical applicability, simplicity, and prognostic accuracy suggest potential for incorporation into multidisciplinary decision‐making, enabling individualized treatment optimization and long‐term outcome improvement.

## Author Contributions

**Zihao Chen:** conceptualization, methodology, software, data curation, investigation, validation, supervision, funding acquisition, visualization, project administration, resources, writing – original draft, writing – review and editing. **Jiangyu Li:** investigation, methodology, writing – original draft. **Ruoxi Gao:** methodology, writing – original draft. **Lei Liang:** data curation, writing – review and editing. **Jun Yang:** funding acquisition, writing – review and editing. **Ting Zhao:** conceptualization, methodology, software, data curation, investigation, validation, writing – review and editing, writing – original draft, resources, project administration, visualization, funding acquisition, supervision. All authors gave final approval of the version to be published; have agreed on the journal to which the article has been submitted; and agree to be accountable for all aspects of the work.

## Declaration of Generative AI and AI‐Assisted Technologies

During the preparation of this work, the authors used Gemini solely for the purpose of improving language, grammar, and readability of the manuscript. Following this utility, the authors thoroughly reviewed, verified, and edited the content as necessary, and take full responsibility for the accuracy and originality of the final publication.

## Ethics Statement

This study was conducted with the approval of the Ethics Committee of The First Affiliated Hospital of Kunming Medical University (Approval Number: 124). All procedures were performed in accordance with the ethical standards of the responsible committee and the Declaration of Helsinki. For this research, informed consent was obtained from all participating individuals.

## Conflicts of Interest

The authors declare no conflicts of interest.

## Supporting information


Supporting File 1

Supporting File 2

## Data Availability

The data of the participants on which the conclusion of this study is based (including the identification information used to support the research results) was provided by Jun Yang according to the licensing agreement and thus cannot be freely disclosed. These data are available upon request. If you wish to obtain these data, please apply to Prof. Jun Yang at the email address: yangjun6@kmmu.edu.cn.

## References

[1] R. L. Siegel , A. N. Giaquinto , and A. Jemal , “Cancer Statistics, 2024,” CA: A Cancer Journal for Clinicians 74, no. 1 (2024): 12–49, 10.3322/caac.21820. 38230766

[2] Y. Wu , J. Ma , X. Yang , et al., “Neutrophil Profiling Illuminates Anti‐Tumor Antigen‐Presenting Potency,” Cell 187, no. 6 (2024): 1422–1439.e24, 10.1016/j.cell.2024.02.005. 38447573

[3] J. Garcia‐Aguilar , S. Patil , M. J. Gollub , et al., “Organ Preservation in Patients With Rectal Adenocarcinoma Treated With Total Neoadjuvant Therapy,” Journal of Clinical Oncology 40, no. 23 (August 2022): 2546–2556, 10.1200/jco.22.00032. PMC9362876 35483010

[4] D. Schrag , Q. Shi , M. R. Weiser , et al., “Preoperative Treatment of Locally Advanced Rectal Cancer,” New England Journal of Medicine 389, no. 4 (2023): 322–334, 10.1056/NEJMoa2303269. PMC10775881 37272534

[5] H. Hu , J. Zhang , Y. Li , et al., “Neoadjuvant Chemotherapy With Oxaliplatin and Fluoropyrimidine Versus Upfront Surgery for Locally Advanced Colon Cancer: The Randomized, Phase III OPTICAL Trial,” Journal of Clinical Oncology 42, no. 25 (September 2024): 2978–2988, 10.1200/jco.23.01889. 38564700

[6] M. J. G. Bond , K. Bolhuis , O. J. L. Loosveld , et al., “First‐Line Systemic Treatment Strategies in Patients With Initially Unresectable Colorectal Cancer Liver Metastases (CAIRO5): An Open‐Label, Multicentre, Randomised, Controlled, Phase 3 Study From the Dutch Colorectal Cancer Group,” Lancet Oncology 24, no. 7 (2023): 757–771, 10.1016/s1470-2045(23)00219-x. 37329889

[7] E. Domingo , C. Kelly , J. Hay , et al., “Prognostic and Predictive Value of Immunoscore in Stage III Colorectal Cancer: Pooled Analysis of Cases From the SCOT and IDEA‐HORG Studies,” Journal of Clinical Oncology 42, no. 18 (2024): 2207–2218, 10.1200/jco.23.01648. PMC11185918 38484206

[8] L. Chen , Y. Wang , C. Cai , et al., “Machine Learning Predicts Oxaliplatin Benefit in Early Colon Cancer,” Journal of Clinical Oncology 42, no. 13 (2024): 1520–1530, 10.1200/jco.23.01080. PMC11095904 38315963

[9] W. A. Hall , J. Li , Y. N. You , et al., “Prospective Correlation of Magnetic Resonance Tumor Regression Grade With Pathologic Outcomes in Total Neoadjuvant Therapy for Rectal Adenocarcinoma,” Journal of Clinical Oncology 41, no. 29 (2023): 4643–4651, 10.1200/jco.22.02525. PMC10564288 37478389

[10] L. Schwarz , J. B. Bachet , A. Meurisse , et al., “Neoadjuvant FOLF(IRIN)OX Chemotherapy for Resectable Pancreatic Adenocarcinoma: A Multicenter Randomized Noncomparative Phase II Trial (PANACHE01 FRENCH08 PRODIGE48 Study),” Journal of Clinical Oncology 43, no. 17 (June 2025): 1984–1996, 10.1200/jco-24-01378. 40184561

[11] C. Swanton , E. Bernard , C. Abbosh , et al., “Embracing Cancer Complexity: Hallmarks of Systemic Disease,” Cell 187, no. 7 (March 2024): 1589–1616, 10.1016/j.cell.2024.02.009. PMC12077170 38552609

[12] S. C. Shah and S. H. Itzkowitz , “Colorectal Cancer in Inflammatory Bowel Disease: Mechanisms and Management,” Gastroenterology 162, no. 3 (2022): 715–730.e3, 10.1053/j.gastro.2021.10.035. PMC9003896 34757143

[13] K. Pelka , M. Hofree , J. H. Chen , et al., “Spatially Organized Multicellular Immune Hubs in Human Colorectal Cancer,” Cell 184, no. 18 (2021): 4734–4752.e20, 10.1016/j.cell.2021.08.003. PMC8772395 34450029

[14] D. Pfister , N. G. Núñez , R. Pinyol , et al., “NASH Limits Anti‐Tumour Surveillance in Immunotherapy‐Treated HCC,” Nature 592, no. 7854 (April 2021): 450–456, 10.1038/s41586-021-03362-0. PMC8046670 33762733

[15] P. T. Ramirez , K. P. Robledo , M. Frumovitz , et al., “LACC Trial: Final Analysis on Overall Survival Comparing Open Versus Minimally Invasive Radical Hysterectomy for Early‐Stage Cervical Cancer,” Journal of Clinical Oncology 42, no. 23 (August 2024): 2741–2746, 10.1200/jco.23.02335. 38810208

[16] B. Chibaudel , M. Raeisi , R. Cohen , et al., “Assessment of the Addition of Oxaliplatin to Fluoropyrimidine‐Based Adjuvant Chemotherapy in Patients With High‐Risk Stage II Colon Cancer: An ACCENT Pooled Analysis,” Journal of Clinical Oncology 42, no. 35 (December 2024): 4187–4195, 10.1200/jco.24.00394. PMC11624096 39231393

[17] K. Ganesh , “Optimizing Immunotherapy for Colorectal Cancer,” Nature Reviews Gastroenterology & Hepatology 19, no. 2 (February 2022): 93–94, 10.1038/s41575-021-00569-4. PMC9487162 34907331

[18] A. M. van der Leun , D. S. Thommen , and T. N. Schumacher , “CD8+ T Cell States in Human Cancer: Insights From Single‐Cell Analysis,” Nature Reviews Cancer 20, no. 4 (2020): 218–232, 10.1038/s41568-019-0235-4. PMC7115982 32024970

[19] R. Bill , P. Wirapati , M. Messemaker , et al., “CXCL9:SPP1 Macrophage Polarity Identifies a Network of Cellular Programs That Control Human Cancers,” Science 381, no. 6657 (2023): 515–524, 10.1126/science.ade2292. PMC10755760 37535729

[20] Z. Chen , L. Liang , C. Pan , et al., “Combined Pan‐Immune‐Inflammation Value and Prognostic Nutritional Index as a Prognostic Biomarker for Colorectal Cancer Undergoing Enterectomy,” Journal of Inflammation Research 18 (2025): 18039–18052, 10.2147/JIR.S570477. PMC12744597 41466854

[21] Z. Rahal , R. El Darzi , S. J. Moghaddam , T. Cascone , and H. Kadara , “Tumour and Microenvironment Crosstalk in NSCLC Progression and Response to Therapy,” Nature Reviews Clinical Oncology 22, no. 7 (2025): 463–482, 10.1038/s41571-025-01021-1. PMC12227073 40379986

[22] L. Cao , S. Zhu , H. Lu , et al., “ *Helicobacter pylori*–Induced RASAL2 Through Activation of Nuclear Factor‐κB Promotes Gastric Tumorigenesis via β‐Catenin Signaling Axis,” Gastroenterology 162, no. 6 (2022): 1716–1731.e17, 10.1053/j.gastro.2022.01.046. PMC9038683 35134322

[23] A. Shafqat , J. A. Khan , A. Y. Alkachem , et al., “How Neutrophils Shape the Immune Response: Reassessing Their Multifaceted Role in Health and Disease,” International Journal of Molecular Sciences 24, no. 24 (2023): 17583, 10.3390/ijms242417583. PMC10744338 38139412

[24] D. J. Propper and F. R. Balkwill , “Harnessing Cytokines and Chemokines for Cancer Therapy,” Nature Reviews Clinical Oncology 19, no. 4 (2022): 237–253, 10.1038/s41571-021-00588-9. 34997230

[25] S. Ghosh , J. Huang , M. Inkman , et al., “Radiation‐Induced Circulating Myeloid‐Derived Suppressor Cells Induce Systemic Lymphopenia After Chemoradiotherapy in Patients With Glioblastoma,” Science Translational Medicine 15, no. 680 (2023): eabn6758, 10.1126/scitranslmed.abn6758. PMC10501302 36696484

[26] N. Verma , P. Kaur , P. Garg , et al., “Clinical and Pathophysiological Characteristics of Non‐Acute Decompensation of Cirrhosis,” Journal of Hepatology 83, no. 3 (September 2025): 670–681, 10.1016/j.jhep.2025.02.028. 40056937

[27] S. Weber and A. L. Gerbes , “COVID‐19 and Liver Injury: Hypoalbuminaemia and γGT Should Be Observed at Hospital Admission,” Gut 72, no. 6 (June 2023): 1229–1230, 10.1136/gutjnl-2022-328214. 35896361

[28] T. Powles , J. W. F. Catto , M. D. Galsky , et al., “Perioperative Durvalumab With Neoadjuvant Chemotherapy in Operable Bladder Cancer,” New England Journal of Medicine 391, no. 19 (November 2024): 1773–1786, 10.1056/NEJMoa2408154. 39282910

[29] K. C. Kao , S. Vilbois , C. H. Tsai , and P. C. Ho , “Metabolic Communication in the Tumour‐Immune Microenvironment,” Nature Cell Biology 24, no. 11 (November 2022): 1574–1583, 10.1038/s41556-022-01002-x. 36229606

[30] A. Tzeng , N. Sangwan , M. Jia , et al., “Human Breast Microbiome Correlates With Prognostic Features and Immunological Signatures in Breast Cancer,” Genome Medicine 13, no. 1 (2021): 60, 10.1186/s13073-021-00874-2. PMC8052771 33863341

[31] E. Urbauer , D. Aguanno , N. Mindermann , et al., “Mitochondrial Perturbation in the Intestine Causes Microbiota‐Dependent Injury and Gene Signatures Discriminative of Inflammatory Disease,” Cell Host & Microbe 32, no. 8 (August 2024): 1347–1364, 10.1016/j.chom.2024.06.013. 39013472

[32] J. Cui , J. Dong , Y. Lang , et al., “Engineered Microalgal Extracellular Vesicles for Enhancing Mitochondrial Homeostasis in Radiodermatitis Prevention,” ACS Nano 19, no. 30 (August 2025): 27634–27653, 10.1021/acsnano.5c07135. 40694433

[33] A. De Leo , A. Ugolini , X. Yu , et al., “Glucose‐Driven Histone Lactylation Promotes the Immunosuppressive Activity of Monocyte‐Derived Macrophages in Glioblastoma,” Immunity 57, no. 5 (2024): 1105–1123.e8, 10.1016/j.immuni.2024.04.006. PMC11114377 38703775

[34] Z. Herold , M. Herold , G. Szentmartoni , et al., “Are Calculated Immune Markers With or Without Comorbidities Good Predictors of Colorectal Cancer Survival? The Results of a Longitudinal Study,” Medical Sciences (Basel, Switzerland) 13, no. 3 (August 2025): 108, 10.3390/medsci13030108. PMC12371976 40843730

