# Supplementary material for: The Combined Value of the Prognostic Nutritional Index and Pan‐Immune‐Inflammation Value in Predicting Neoadjuvant Chemotherapy and Survival in Locally Advanced Colon Cancer: A Retrospective Study
Source: Immun Inflamm Dis. 2026 Nov 5;14(11):e70572. doi: 10.1002/iid3.70572 (PMC13737460; doi:10.1002/iid3.70572)
Supplement: Supplementary file 2 [file IID3-14-e70572-s002.docx]

Table S1 The clinical characteristics of 241 patients with locally advanced colon cancer

| **Characteristics** | **Case (%)** | **Mean (SD)** | **Range** |
| --- | --- | --- | --- |
| **Gender** |  |  |  |
| Male | 163(67.6) |  |  |
| Female | 78(32.4) |  |  |
| **Age (years)** |  | 59.02**±**11.25 | 24-83 |
| ≤ 60 | 128(53.1) |  |  |
| > 60 | 113(46.9) |  |  |
| **BMI (Kg/m^2^)** |  | 22.47**±**3.13 | 14.57-30.80 |
| ≤ 25 | 199(82.6) |  |  |
| > 25 | 42(17.4) |  |  |
| **Minority ethnic group** |  |  |  |
| Yes | 21(8.7) |  |  |
| No | 220(91.3) |  |  |
| **MMR status** |  |  |  |
| pMMR | 211(87.6) |  |  |
| dMMR | 30(12.4) |  |  |
| **Tumor size (cm)** |  | 2.78**±**1.83 | 0.1-13.0 |
| < 5.0 | 209(86.7) |  |  |
| ≥ 5.0 | 32(13.3) |  |  |
| **cT stage** |  |  |  |
| T2 | 95(39.4) |  |  |
| T3 | 76(31.5) |  |  |
| T4 | 70(29.1) |  |  |
| **cN stage** |  |  |  |
| N0 | 41(17.0) |  |  |
| N1 | 181(75.1) |  |  |
| N2 | 19(7.9) |  |  |
| **cTNM stage** |  |  |  |
| Ⅱ | 74(30.7) |  |  |
| Ⅲ | 167(69.3) |  |  |
| **Chemotherapy regimen** |  |  |  |
| CAPEOX | 122(50.6) |  |  |
| FOLFOX | 119(49.4) |  |  |
| **Surgical Procedures** |  |  |  |
| Laparoscopic | 15(6.2) |  |  |
| Laparotomy | 226(93.8) |  |  |
| **Pre-treatment PIV** **(**$\bar{\boldsymbol{X}}\boldsymbol{\pm S}$**)** |  | 380.71**±**645.63 | 27.8-2636.7 |
| **Pre-treatment PNI (**$\bar{\boldsymbol{X}}\boldsymbol{\pm S}$**)** |  | 51.4**±**8.8 | 31.7-81.1 |

**Abbreviations:** PIV, Pan-immune-inflammation value; PNI, Prognostic nutritional index; pMMR: Proficient mismatch repair‌; dMMR: Deficient mismatch repair‌.

Table S2 The association between clinicopathological features and PIV-PNI score

**Abbreviations:** PIV, Pan-immune-inflammation value; PNI, Prognostic nutritional index; pMMR: Proficient mismatch repair‌; dMMR: Deficient mismatch repair‌.

| **Variables** | **PIV-PNI** | | | ***p value*** |
| --- | --- | --- | --- | --- |
|  | **0 score (n=72)** | **1 score (n=96)** | **2 score (n=73)** |  |
| **Gender** |  |  |  | 0.8279 |
| Male | 48(66.7%) | 67(69.8%) | 48(65.8%) |  |
| Female | 24(33.3%) | 29(30.2%) | 25(34.2%) |  |
| **Age (years)** |  |  |  | 0.3682 |
| ≤ 60 | 34(47.2%) | 51(53.1%) | 43(58.9%) |  |
| > 60 | 38(52.7%) | 45(46.9%) | 30(41.1%) |  |
| **Minority ethnic group** |  |  |  | 0.8440 |
| Yes | 7(9.7%) | 9(9.4%) | 5(6.8%) |  |
| No | 65(90.2%) | 87(90.6%) | 68(93.2%) |  |
| **MMR status** |  |  |  | 0.5212 |
| pMMR | 69(95.8%) | 92(95.8%) | 67(91.8%) |  |
| dMMR | 3(4.2%) | 4(4.2%) | 6(8.2%) |  |
| **Tumor size (cm)** |  |  |  | 0.0588 |
| < 5.0 | 67(93.1%) | 84(87.5%) | 58(79.5%) |  |
| ≥ 5.0 | 5(6.9%) | 12(12.5%) | 15(20.5%) |  |
| **Infiltrating depth** |  |  |  | 0.0078 |
| T2 | 32(44.5%) | 32(33.3%) | 31(42.5%) |  |
| T3 | 19(26.4%) | 31(32.4%) | 26(35.6%) |  |
| T4 | 21(29.1%) | 33(34.3%) | 16(21.9%) |  |
| **Lymph node** **metastasis** |  |  |  | 0.2158 |
| N1 | 5(6.9%) | 17(17.7%) | 9(9.3%) |  |
| N0 | 65(90.3%) | 73(76.0%) | 61(83.6%) |  |
| N2 | 2(2.8%) | 6(6.3%) | 3(3.1%) |  |
| **Clinical stage** |  |  |  | 0.1350 |
| Ⅱ | 56(77.8%) | 61(63.5%) | 50(68.5%) |  |
| Ⅲ | 16(22.2%) | 35(36.5%) | 23(31.5%) |  |
| **Differentiation degree** |  |  |  | 0.0117 |
| Poorly | 66(91.7%) | 86(89.6%) | 55(75.3%) |  |
| Well-Moderately | 6(8.3%) | 10(10.4%) | 18(24.7%) |  |
| **Surgical Procedures** |  |  |  | 0.0031 |
| Laparoscopic | 0(0%) | 6(6.3%) | 9(12.3%) |  |
| Laparotomy | 72(100.0%) | 90(93.7%) | 64(87.7%) |  |
| **Chemotherapy regimen** |  |  |  | 0.9999 |
| CAPEOX | 36(50.0%) | 49(51.0%) | 37(50.7%) |  |
| FOLFOX | 36(50.0%) | 47(49.0%) | 36(49.3%) |  |

Table S3 Relationship between tumor response and the PIV-PNI score

**Abbreviations:** PIV, Pan-immune-inflammation value; PNI, Prognostic nutritional index; ORR, Objective response rate; DCR, Disease control rate; TRG: Tumor regression grade.

| **Tumor response** | **PIV-PNI score (%)** | | | ***p value*** |
| --- | --- | --- | --- | --- |
| **RECIST version 1.1 evaluation**^a^ | 0(n=58) | 1(n=41) | 2(n=29) |  |
| ORR | 50(86.2) | 24(58.3) | 11(37.9) |  |
| DCR | 52(89.7) | 29(70.7) | 26(89.7) |  |
| **Pathologic TRG grading**^b^ |  |  |  | <0.0001 |
| TRG3 (n=40) | 1(1.4) | 10(10.4) | 29(39.7) |  |
| Non-TRG3 (n=201) | 71(98.6) | 86(89.6) | 44(60.3) |  |

**Note:** ORR=(CR+PR)/(CR+PR+SD+PD); DCR=(CR+PR+SD)/(CR+PR+SD+PD). CR is defined as the disappearance of all target lesions.PR is defined as a reduction of at least 30% in the sum of the target lesion diameters from the sum of the baseline diameters. PD is defined as a minimum increase in the sum of the target lesion diameters of at least 20% relative to the smallest sum of the diameters in the study.SD is defined as neither sufficient reduction to qualify for PR nor sufficient increase to qualify for PD. The target lesion was a measurable lymph node lesion with a short diameter of >1.5cm. The change in the sum of the diameters of all target lesions before and after neoadjuvant therapy was used to calculate the efficacy evaluation. a, evaluation of measurable lymph node lesions (short diameter>1.5cm) before and after neoadjuvant therapy; b, evaluation of primary lesions before and after neoadjuvant therapy
